# Liver fibrosis is strongly associated with an enhanced level of immunosuppressive tryptophan catabolism independently of HCV viremia in ART-treated HIV/HCV co-infected patients

**DOI:** 10.1186/1471-2334-14-S2-O16

**Published:** 2014-05-23

**Authors:** Mohammad-Ali Jenabian, Ido Kema, Robert Paulino Ramirez, Sahar Saeed, Kathleen Rollet, Kishanda Vyboh, Jean-Carlos Tejada, Norbert Gilmore, Marina B Klein, Jean-Pierre Routy

**Affiliations:** 1 Chronic Viral Illnesses Service of the McGill University Health Center, Montreal, Quebec, Canada; 2 Research Institute of the McGill University Health Center, Montreal, Quebec, Canada; 3 Department of Laboratory Medicine, University Medical Center, Groningen, University of Groningen, The Netherlands; 4 School of Medicine, Research Department, Universidad Iberoamericana, Santo Domingo, Dominican Republic; 5 Clinical Research Solutions, Montreal, Quebec, Canada Division of Hematology, McGill University Health Center, Montreal, Quebec, Canada; 6 Instituto Dominicano de Estudios Virologicos, Santo Domingo, Dominican Republic

## Background

HCV infection induces hepatic and extra-hepatic damage that includes kidney and neurocognitive dysfunction. Tryptophan (Trp) is catabolized into immunosuppressive kynurenine (Kyn) by indoleamine 2,3-dioxygenase (IDO) and tryptophan 2,3 dioxegenase (TDO). Increased Trp catabolism measured by Kyn/Trp ratio has been associated with neurocognitive impairment and immune dysfunction in HIV mono-infection. Here, we assessed the contribution of Trp catabolism in HCV/HIV co-infected patients.

## Methods

Plasma samples were collected from ART-treated (HIV RNA <40 copies/ml) HCV/HIV co-infected patients with or without liver fibrosis (n=20 per group), HBV/HIV co-infected patients (n=25), ART-treated and untreated HIV-mono-infected patients and 30 healthy subjects (HS), (n=30 per group). Furthermore, 17 additional HCV/HIV INF-α/ribavirin treated patients were longitudinally assessed before and 6 months after sustained virological response (SVR). IDO and TDO enzymatic activity (Kyn/Trp ratio) was measured by isotope dilution tandem mass spectrometry. Statistical analyses were performed using Anova, unpaired or paired t-tests and Spearman correlation tests.

## Results

Among HCV/HIV patients, those having fibrosis compared with non-fibrosis had higher APRI scores (2.48±0.23 vs 0.36±0.018, p<0.0001) and elevated Kyn levels (2.6±0.24 vs. 1.97±0.15 µmol/L, p=0.038). For HBV/HIV co-infected, Kyn level was also elevated (2.1±0.16 µmol/L). The Kyn/Trp ratio was equally elevated in all HCV and HBV co-infected groups, similar to the untreated mono-infected HIV group. Importantly, HCV/HIV fibrotic and HBV/HIV groups but not the non-fibrotic group had higher Kyn/Trp ratios compared to the ART-treated and HS groups. Unlike HIV viremia, HCV viremia was not correlated with the Kyn/Trp ratio. However, in all HCV/HIV co-infected patients, Kyn/Trp ratio was correlated with the APRI score (p=0.027). Successful HCV treatment improved APRI score (0.89±0.13 vs. 0.4±0.04, p=0.001), contrasting with unchanged elevated Kyn/Trp ratios six months after SVR.

## Conclusion

ART-treated HCV/HIV and HBV/HIV co-infected patients presented with elevated immunosuppressive Kyn/Trp ratios when compared to mono-infected HIV-treated patients and reached a ratio similar to the untreated HIV mono-infected patients. In ART-treated patients, liver fibrosis on its own, but not HCV viremia, was associated with an enhanced level of immunosuppressive Tryptophan catabolism. These findings suggest that a necrotico-inflammatory liver syndrome persists even after SVR, and subsequently induces a systemic immune activation by increasing tryptophan catabolism.

